# Risk prevalence, readiness and confidence to change lifestyle risk factors among clients of community mental health services

**DOI:** 10.1177/00048674241257751

**Published:** 2024-06-06

**Authors:** Tegan Stettaford, Caitlin Fehily, Elizabeth Campbell, Daniel Barker, Christopher Oldmeadow, Emma McKeon, Sophie Love, Sharon Lawn, David Castle, Jennifer Bowman

**Affiliations:** 1 School of Psychological Sciences, The University of Newcastle, Callaghan, NSW, Australia; 2 Hunter Medical Research Institute, Clinical Research Centre, New Lambton Heights, NSW, Australia; 3 School of Medicine and Public Health, The University of Newcastle, Callaghan, NSW, Australia; 4 Hunter New England Population Health, Wallsend, NSW, Australia; 5 Lived Experience Australia, Brighton, SA, Australia; 6 College of Medicine and Public Health, Flinders University, Adelaide, SA, Australia; 7 School of Psychological Sciences, University of Tasmania, Hobart, TAS, Australia; 8 Tasmanian Centre for Mental Health Service Innovation, Hobart, TAS, Australia

**Keywords:** Confidence, community mental health services, lifestyle risk factors, mental health, readiness

## Abstract

**Objective:**

People with mental health conditions have high rates of chronic physical diseases, partially attributable to lifestyle risks factors. This study examined risk prevalence among community mental health service clients, their readiness and confidence to change, and associations with participant characteristics.

**Methods:**

Cross-sectional survey of adult clients from 12 community mental health services across 3 local health districts in New South Wales, Australia, collected from 2021 to 2022. Participants (*n* = 486) completed a telephone interview determining five risk factors, and readiness and confidence to change these. Multiple binary logistic regression models determined associations between readiness and confidence (for each risk), and participant characteristics (demographics and diagnosis).

**Results:**

Participants most commonly reported a diagnosis of schizophrenia (36.7%) or depression (21.1%). Risk factors were prevalent: ranging from 26% (harmful alcohol use) to 97% (poor nutrition). High readiness was greatest for smoking (68%), weight (66%) and physical inactivity (63%), while confidence was highest for changing alcohol use (67%). Two significant associations were identified; females were more likely than males to have high readiness to change nutrition (odds ratio = 1.14, confidence interval = [1.13, 2.34], *p* = 0.0092), with males more likely to have high confidence to change physical activity (odds ratio = 0.91, confidence interval = [0.45, 0.99], *p* = 0.0109).

**Conclusions:**

Many participants were ready and confident to change risk factors. Gender influenced readiness to change nutrition and physical activity confidence. Training to upskill mental health clinicians in provision of preventive care that builds confidence and readiness levels may aid in supporting positive behaviour change.

## Introduction

Systematic review evidence notes life expectancy is a median of 15 years lower for people with mental health conditions than the general population (Walker et al., 2015). In Australia, it has been reported that 78% of the excess mortality experienced by people with mental health conditions is due to higher levels of chronic diseases (Lawrence et al., 2013), including heart and respiratory diseases, with similar reports internationally (Colton and Manderscheid, 2006; Liu et al., 2017; Walker et al., 2015).

Globally, modifiable lifestyle risk factors – including smoking, poor nutrition, alcohol over-consumption, physical inactivity and overweight/obesity (SNAPW) (IHME, 2019; Smith et al., 2020) – are associated with preventable chronic disease morbidity and mortality (Firth et al., 2019). These factors are more common among people with mental health conditions than those without (Bartlem et al., 2015; Lawrence et al., 2013), with the largest and most consistent difference being for smoking (Drope et al., 2018; Richardson et al., 2019; Stockings et al., 2013). People with a mental health condition are also more likely to have multiple risk factors (Bartlem et al., 2015; Champion et al., 2018; Regan et al., 2022; Stanley and Laugharne, 2014).

In Australia, government-funded community mental health services (CMHS) are a key provider of outpatient mental health care, seeing approximately 468,800 clients annually for brief (1–14 days) to long-term treatment (92+ days), both voluntary and involuntary (AIHW, 2023). CMHS clients have a range of diagnoses, including serious mental illnesses (SMI), and span across all age groups and metropolitan and rural areas (AIHW, 2022, 2023). Policy and clinical practice guidelines acknowledge the need for mental health services, including CMHS, to address lifestyle risk factors (AIHW, 2022; Das et al., 2016; NSW Health, 2017; RANZCP, 2015), yet this is not being implemented successfully (Kaine et al., 2022; Lambert et al., 2017). In order to inform initiatives to support the implementation of preventive care guidelines in CMHS, it is necessary to understand more about the risk profiles and interest in behaviour change specifically among clients accessing such services. A setting-specific approach is particularly important, given previous research has found some differences across mental health settings (e.g. inpatient vs community) in chronic disease profiles (Walker et al., 2015) and prevalence of risk behaviours (Cook et al., 2014; de Oliveira et al., 2020).

Two previous studies examined the prevalence of multiple SNAPW risks in clients of Australian CMHS (Bartlem et al., 2015; Regan et al., 2022). Bartlem et al.’s (2015) survey of 558 participants from 12 CMHS within one health district in New South Wales (NSW), Australia, reported prevalence of SNAP risks against Australian guidelines, ranging from 43.2% (alcohol over-consumption) to 86.7% (insufficient fruit/vegetable consumption). 78.4% of participants were at risk for two or more factors, with 10.2% at risk for all. More recently, Regan et al. (2022) surveyed 567 participants from 2 CMHS from the same NSW health district. The proportions with SNAPW risks were again high, ranging from 20% (chronic alcohol risk) to 82% (strength training).

To help understand how clients may respond to lifestyle behaviour change support from CMHS and their likelihood of achieving change, factors such as readiness to change and confidence in making changes are important, as they are linked to behaviour change success (Abar et al., 2013; Bertholet et al., 2012). Measurement of these constructs has varied across previous literature. For instance, the ‘stages of change’ model describes 6 stages of readiness to change: pre-contemplation, contemplation, preparation, action, maintenance and relapse/termination (Prochaska and Velicer, 1997). Confidence is typically assessed through Likert-type scales or ratings (e.g. 1–10), aligning with counselling techniques to support behaviour change (Bailey, 2019; Bodenheimer et al., 2007; Hladek et al., 2020). No previous studies were identified that measured either of these constructs in Australian CMHS clients, though international studies in other mental health settings have assessed client readiness (Apodaca et al., 2007; Lee et al., 2018; Metse et al., 2016; Prochaska et al., 2014; Stockings et al., 2013), and/or confidence to change (Mann-Wrobel et al., 2011; Twyford and Lusher, 2016; Tzilos et al., 2014) for at least one SNAPW risk. Higher readiness levels have been linked to positive behaviour change for physical activity and smoking, among people with mental health conditions (Farholm and Sørensen, 2016; Stockings et al., 2013; Tzilos et al., 2014), while higher confidence has been shown to be associated with behaviour change for smoking and physical activity (Apodaca et al., 2007; Twyford and Lusher, 2016).

Regarding other mental health settings, international studies within psychiatric inpatient populations have reported high levels of readiness to stop smoking ranging from 28.8% to 45% (Apodaca et al., 2007; Metse et al., 2016; Stockings et al., 2013), aligning with at least ‘contemplative’ within the stages of change model (Prochaska and Velicer, 1997). Siru et al. (2009) reviewed 29 studies assessing readiness to change smoking via stages of change (Prochaska and Velicer, 1997) measures, with 9 of these studies focusing on psychiatric populations with the remaining 20 in general populations for comparison. Smokers with a mental health condition had higher rates of readiness than those without; 38% (vs 33%) in contemplation and 19% (vs 10%) in preparation. Prochaska et al. (2014) considered readiness across multiple SNAP risk factors, measuring readiness according to the stages of change model among a sample of acute inpatient psychiatric unit clients. Nearly a quarter of smokers (23%) were classified as prepared, with even higher rates for other risks: 46% for fruit and vegetable intake, 51% for physical inactivity and 57% for binge drinking.

Several studies in other mental health settings have examined confidence to quit smoking among people with mental health conditions (Apodaca et al., 2007; Prochaska et al., 2014; Tzilos et al., 2014). One study among inpatient adolescents measured confidence to change smoking on an 11-point scale (Tzilos et al., 2014), from ‘not at all confident’ to ‘very confident’. Low confidence was reported (average, 4.9), with confidence significantly associated with intention to quit. Similarly, a study among adults with schizophrenia reported low levels of confidence to quit smoking, with the majority either not at all, a little or moderately confident (15%, 32.5% or 30%, respectively) (Mann-Wrobel et al., 2011). With regard to other risk factors, one previous review examined motivation for physical activity among people with SMI in inpatient and outpatient settings; finding that some included studies mentioned low confidence as a barrier to physical activity (Farholm and Sørensen, 2016).

We found no studies in Australian CMHS examining associations between readiness or confidence to change factors and participant characteristics. Regarding physical activity among people with SMI, a previous review exploring associations between characteristics (body mass index [BMI], gender, age, diagnosis, medication) and psychosocial predictors found no associations with readiness, while gender was associated with self-efficacy (favouring men) (Farholm and Sørensen, 2016).

As research has not explored Australian CMHS clients’ readiness and confidence for risk factor change, this study examined the prevalence of and readiness and confidence to change risk factors (SNAPW) among a sample of CMHS clients with a broad range of mental health conditions. We also assessed associations between readiness and confidence, and participant characteristics (demographics and mental health diagnosis), to enhance understanding of factors potentially influencing lifestyle change.

## Method

### Design and setting

A cross-sectional survey of clients of 12 CMHS was undertaken using computer-assisted telephone interviews (CATIs) from September 2021 until February 2022. Data were collected at the baseline of a cluster randomised controlled trial (Fehily et al., 2022). The 12 CMHS spanned 3 local health districts (LHDs) within NSW, Australia (6 CMHS in 1 LHD, 3 in each of the other two). Mental health care within these services is generally provided through consultations (of varying number and frequency) with an allocated clinician. CMHS see clients with a variety of diagnoses and severities, including Schizophrenia (21%), depressive episodes (6%) and bipolar disorder (5%) (AIHW, 2024b).

### Participants and recruitment

#### Community mental health services

Services were deemed eligible to participate if they were not currently involved in any other research with the team. Twelve eligible, discrete services were identified after assessment of management structure (a single manager), size (number of clinical staff, annual client throughput) and site structure (multi-site or single site, with smaller sites with a common manager considered one service) (Fehily et al., 2022). Of the 12 services involved, 2 comprised smaller individual services (each with 3 locations). Services span Major Cities (*n* = 4), Inner Regional Australia (*n* = 7) and Outer Regional Australia (*n* = 5), according to the 2016 ASGC Remoteness Area Classification (ABS, 2018). All CMHS agreed to participate.

#### Participants

Clients were eligible to participate in the survey if they were 18 years or older, had attended at least two appointments with their CMHS in the previous 9 months and had a valid phone number and mailing address. Clients meeting eligibility criteria were identified by an independent health service statistician using data from electronic medical records. Over 3 months, the statistician randomly selected 1800 clients from the eligible pool of approximately 4600 (total pool approximately 6250) (889 in LHD 1, 565 in LHD 2, 346 in LHD 3; average, 150 per service). These participants were invited via a mailed information letter from their CMHS informing them of the survey and providing opportunity to opt-out by calling a toll-free number.

Those who did not opt out were called by trained NSW Health service CATI interviewers approximately 2 weeks following the letter to determine additional eligibility criteria: English speaking, physically and mentally capable of taking part, and not having an eating disorder as their primary diagnosis. Consenting participants were administered the survey and received a $10 gift card.

### Data collection and procedures

Data obtained from participants’ electronic medical records included LHD, service attended, age and postcode. Other participant characteristics were obtained during the survey. Interviewers were blind to service allocation. Data were recorded in REDCap. The survey took on average 25 minutes.

### Measures

#### Participant characteristics

Age and postcode (for Socio-Economic Indexes of Areas [SEIFA] and Accessibility/Remoteness Index of Australia [ARIA] classifications) (ABS, 2021, 2023) were gathered from electronic records. Participants self-reported their gender, Indigenous identification, primary mental health condition, pregnancy status (if not identifying as male), employment status, marital status, highest level of achieved education, height and indicated from a list any physical conditions for which they receive medical care.

#### Participant risk variables

Participants self-reported their current engagement in lifestyle risks (SNAPW) to enable comparison to Australian National Guidelines. Survey questions were adapted from previous research by the team and validated tools including the International Physical Activity Questionnaire (IPAQ), Alcohol Use Disorders Identifications Test (AUDIT-C) and BMI (see Table 1).

**Table 1.** Lifestyle risk measures and national guidelines.

Lifestyle risk factor	Measures [*response options*]	Definition of risk (according to national guidelines)
Smoking	Do you currently smoke any cigarettes? [*yes, daily; yes, at least once a week; yes, less than once a week; not at all (quit less than 6* *months ago); not at all (quit 6* *months or more ago); not at all (never smoked)*]	Any level of tobacco smoking (Australian Government Department of Health, 2019)
Overall nutrition (fruit and/or vegetable consumption)	Thinking about the last month, how many serves of fruit did you usually eat each day? [*Open ended*] Thinking about the last month, how many serves of vegetables did you usually eat each day? [*Open ended*]	Consuming less than 2 serves of fruit and/or 5 serves of vegetables per day (NHMRC, 2013)
Overall alcohol (acute and/or chronic)	Thinking of the last month, how often did you consume a drink containing alcohol? [N*ever; I don’t drink (e.g. never have; ceased drinking; not currently drinking); None in the last month (e.g. nil last month; drinks but less than monthly); Once a month; 2 to 4 times a month; 2 to 3 times a week; 4 or more times a week*] In a typical week, how many standard drinks did you consume? A standard drink is 1 schooner of light beer, a middy of full-strength beer, a 100 mL glass of wine, or a 30 mL nip of spirits. [*Open ended*] Thinking of the last month, how often did you consume 5 or more standard drinks on any one day? [*Never; Not in the last month; Monthly; Weekly; Daily or almost daily*] (Derived from the AUDIT-C; Babor et al., 2001)	Drinking more than 10 standard drinks in a week (chronic harm), and/or more than 4 on one occasion (acute harm) (NHMRC, 2020)
Overall physical activity (moderate/vigorous minutes and/or strength training)^a^	In a typical week, how many times did you do vigorous/moderate physical activities? [*Open ended*] On average, how many minutes per session did you spend on those vigorous/moderate physical activities? [*Open ended*] In a typical week, how many days did you do any type of muscle strengthening activities such as exercise using free weights, body weight exercises or gym-based strength exercises? [*Open ended*] On average, how many minutes per day did you spend strength training? [*Open ended*] (Derived from the IPAQ; Craig et al., 2003)	Engaging in less than 2.5 to 5 hours of moderate or 1.25 to 2.5 hours of vigorous physical activity (or equivalent combination) and/or less than 2 days of strength training in a week (Australian Government Department of Health, 2021)
Weight	Could you tell me your height in centimetres? [*Open ended*] Could you please tell me your current weight in kilograms? [*Open ended*]	BMI > 24.9 (NHMRC, 2013)

a Participants were provided examples of the different levels of physical activity: ‘VIGOROUS physical activity, like running, jogging, gym classes, boxing, soccer or squash, that makes you breathe harder or puff. During those activities it is hard to have a conversation . . . MODERATE physical activity – which requires some effort, but you can also hold a conversation. Activities like fast or brisk walking, baseball, tennis, easy bicycling, volleyball and easy swimming. This doesn’t include easy walking to travel from place to place, and for recreation’.

#### Participant readiness and confidence to change

To measure readiness to change, the readiness ruler (Gordon et al., 2010; Heather et al., 2008) – originally developed for alcohol use, with responses based on the stages of change model – was adapted for SNAPW factors. Per factor, participants were asked to select from 1 to 5: I never think of changing [factor]; I sometimes think about changing [factor]; I have decided to change [factor]; I am already trying to change [factor]; my [factor] has changed (Heather et al., 2008). Participants responded to a 1–10 scale, asking how confident they were in changing each factor (1, not at all and 10, extremely confident) (Bailey, 2019; Bodenheimer et al., 2007; Hladek et al., 2020).

Readiness and confidence questions around smoking and alcohol consumption were only asked if someone indicated they were a smoker, or if they reported any alcohol consumption, respectively.

### Statistical analysis

SAS (v9.4, *Cary, North Carolina*) was utilised for data management and analyses.

#### Variable transformation

Missing or ‘don’t know’ responses were treated as missing. Participant characteristic variables were condensed (see Table 2). Postcodes were used to calculate ARIA (remoteness) and SEIFA (index of disadvantage) (ABS, 2006). Physical activity data were cleaned in line with the IPAQ Guidelines (Forde, 2005).

**Table 2.** Characteristics of the 486 clients who participated in the survey.

Variable	*n/N*	%
Gender		
Male	223/486	45.8
Female	257/486	52.8
Transgender or gender non-conforming	4/486	0.8
Other	2/486	0.4
Age		
18–34	138/486	28.4
35–54	253/486	52.1
55+	95/486	19.5
Employment status		
Employed	122/486	25.1
Unemployed	153/486	31.4
Unable to work due to health reasons	131/486	26.9
Other^a^	80/486	16.4
Marital status		
Never married	269/485	55.4
Married or living together in a relationship	121/485	24.9
Other^b^	95/485	19.5
Highest level of education completed		
Some high school or less	182/485	37.5
Completed high school certificate	82/485	16.9
TAFE certificate or diploma	157/485	32.3
University degree or higher	64/485	13.2
Aboriginal and/or Torres Strait Islander	70/483	14.4
Index of disadvantage (ABS, 2023)^c^		
Lower SES	414/485	85.3
Higher SES	71/485	14.6
Remoteness (ABS, 2021)		
Major Cities of Australia	170/485	35.1
Inner Regional Australia	234/485	48.2
Outer Regional Australia	81/485	16.7
Primary mental health condition		
Schizophrenia and other psychotic disorders	170/463	36.7
Depression	98/463	21.1
Post-traumatic stress disorder (PTSD)	54/463	11.6
Bipolar disorder	52/463	11.2
Anxiety disorder	33/463	7.1
Personality disorder	28/463	6.1
Substance use disorder	8/463	1.7
Other^d^	20/463	4.3
Pregnant^e^	8/262	3.0
Current physical health condition requiring medical treatment (any)	224/486	46.1
Medical condition receiving care for		
Heart disease	12/486	2.4
Stroke	1/486	0.2
High blood pressure	28/486	5.7
High blood cholesterol	13/486	2.6
Blood clot	2/486	0.4
Diabetes	47/486	9.6
Osteoporosis	4/486	0.8
Cancer	7/486	1.4
Asthma	37/486	7.6
Other lung or respiratory condition	9/486	1.8
Arthritis	23/486	4.7
Overweight/obesity	9/486	1.8
Other^f^	150/486	30.8

*n*’s for response options do not always add to 486 due to missing responses (e.g. if participants responded don’t know or prefer not to say).

a Includes students, carers, volunteers and retirees.

b Includes those separated, divorced and widowed.

c ⩽990 was coded as high disadvantage; based on NSW median.

d Other disorders reported include obsessive-compulsive disorder, ADHD and brain injury.

e Not asked of participants who identified as male.

f Other conditions reported include sleep apnoea, spinal injury/disorder and low blood pressure.

Participants were coded as highly ready if they selected an option from 3 (I have decided to change [factor]) through 5 (my [factor] has changed) for readiness, and highly confident if they rated themselves from 7 to 10 (Bailey, 2019; Bodenheimer et al., 2007; Hladek et al., 2020).

BMI was calculated as weight ÷ height^2^ (NHMRC, 2013).

#### Approach to analysis

Descriptive statistics were used to summarise participant characteristics, and the prevalence, readiness and confidence levels for SNAPW. Chi-squares were undertaken to compare eligible consenters and non-consenters on age, ARIA and SEIFA.

To assess associations between readiness and confidence for each SNAPW factor (10 dependent variables) and participant characteristics (7 independent variables: gender, age, employment status, marital status, education, remoteness, mental health condition). Bivariate cross-tabulations were first conducted to determine any variables with insufficient sample sizes (cell count less than 5) to be excluded. Multiple logistic regression was then employed, using three methods: Least Absolute Shrinkage and Selection Operator (LASSO), forward selection and backward selection. Results from the LASSO models are reported, given the procedure uses a stricter penalty-based method with results from the backward and forward models (more lenient test-based selection methods) included in Supplementary Files 2 and 3 to provide additional supportive evidence. The Akaike information criterion (AIC) was used to guide variable selection. The significance level was α = 0.05. Odds ratios (ORs) and their respective 95% confidence intervals (CIs) and *p*-values were obtained from the models. Multiplicity adjustments to control the family-wise type 1 error rate were not considered due to the exploratory nature of the work.

### Ethics

This study was approved by the Hunter New England Human Research Ethics Committee (Ref: 2020/ETH03234) and the University of Newcastle Human Research Ethics Committee (Ref: H-2021-015), as well as being authorised for conduct by the Central Coast (Ref: 2021/STE00265) and Mid North Coast (Ref: 2021/STE00264). Informed consent was obtained from all participants involved.

## Results

Supplementary File 1 summarises recruitment: 1800 clients were invited to participate, of whom 21 opted out of further contact about the study. Of the remaining 1779, 68% were able to be contacted by interviewers and, of these, 4% were ineligible, 56% declined, with 40% completing the survey and providing data for this study (*n* = 486/1219). Consenters were significantly more likely to be of lower socioeconomic status (SES), compared to non-consenters (*p* = 0.04), with no significant differences for age (*p* = 0.21) or remoteness (*p* = 0.23). Each participating service contributed between 24 and 69 clients. Table 2 shows participant characteristics.

### Prevalence of lifestyle risk factors

As shown in Table 3, almost all participants (96.8%) were at risk for poor nutrition, most (86.6%) were physically inactive, and around 70% were overweight/obese. Nearly half (44.8%) were current smokers and about a quarter (26.2%) were drinking alcohol above guidelines.

**Table 3.** Prevalence of risk factors among the 486 participants.

Lifestyle factor	*n/N*	%
Smoking	217/484	44.8
Overall nutrition^a^	468/483	96.8
Inadequate fruit	336/483	69.5
Inadequate vegetables	449/477	94.1
Overall alcohol^a,b^	124/472	26.2
Chronic consumption	56/469	11.9
Acute consumption	116/472	24.5
Overall physical activity^a,c^	420/485	86.6
Inadequate moderate/vigorous physical activity	326/482	67.6
Inadequate strength training	368/486	75.7
Overall BMI (overweight/obese)^d^	270/379	71.2
Underweight	17/379	4.4
Healthy weight	92/379	24.2
Overweight^e^	97/379	25.5
Obese^e^	173/379	45.6

a People deemed at risk if: (1) they are at risk for both levels or (2) they are at risk for one, and not/missing the other. Classed as not at risk if data for both levels indicates no risk.

b Participants indicating pregnancy were removed.

c One participant was noted to have a value for physical activity outside of the cut-off scores as per the IPAQ Guidelines, which was treated as missing.

d BMI was only calculated if there was data for both weight and height, hence missing data. Participants indicating pregnancy were also removed.

e Defined as ‘at risk’ for this study.

### Readiness and confidence to change lifestyle risk factors

**Graph 1.**
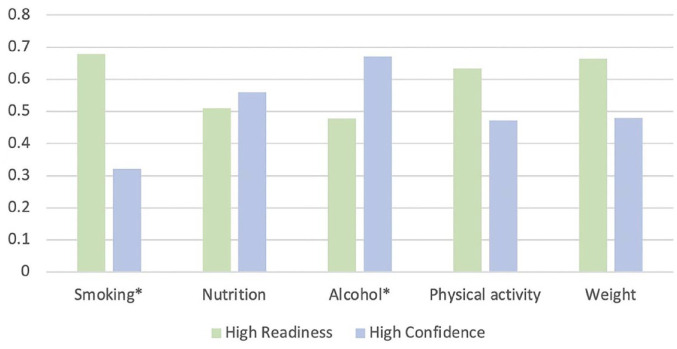
Proportion of participants with high confidence and readiness to change. *Only asked of participants who indicated that they smoked or consumed alcohol, respectively.

The proportions of participants with high readiness and high confidence to change each risk factor are shown in Table 4 and Graph 1. At least half of the respondents were highly ready to change smoking, nutrition, physical activity and weight, with the highest proportion for smoking (68%). At least half of the respondents reported high confidence to change nutrition and over two-thirds, alcohol. The lowest proportion with high confidence was for smoking (32%).

**Table 4.** Prevalence of high readiness and confidence per lifestyle risk factor.

Lifestyle factor	High readiness to change^a^		High confidence to change^b^	
	*n/N*	%	*n/N*	%
Smoking^c^	146/215	67.9	67/209	32.1
Nutrition (fruit and/or vegetables)	242/475	50.9	261/466	56.0
Alcohol^c^	137/286	47.9	184/274	67.1
Physical activity	300/473	63.4	225/477	47.1
Weight (BMI)	313/471	66.4	222/462	48.1

a Participants rating their readiness as either: I have decided to change [factor]; I am already trying to change [factor]; my [factor] has changed.

b Participants rating their confidence as 7–10 on a 1–10 scale were categorised as having high confidence.

c Only asked of participants who indicated that they smoked or consumed any alcohol, respectively.

For smoking, physical activity and weight, the proportion of participants reporting high readiness was larger than the proportion reporting high confidence. For alcohol and to a smaller extent nutrition, the proportion reporting high confidence was larger than the proportion reporting high readiness (Graph 1).

### Associations between readiness and confidence to change lifestyle risk factors and participant characteristics

Table 5 provides the significant results from the LASSO models exploring associations between participant characteristics and readiness and confidence to change SNAPW (results from forward and backward models are presented in Supplementary Files 2 and 3). Of the 10 models conducted, 2 retained independent variables: readiness to change nutrition and confidence to change physical activity.

**Table 5.** Results of LASSO models for confidence and readiness to change.

Variable	AIC	Odds ratio	Lower CI	Upper CI	*p*-value
Readiness to change nutrition					
Gender					
Female	619.98	1.135627	1.127497	2.342222	0.0092*
Male		1			
Confidence to change physical activity					
Gender					
Female	618.966	0.912367	0.432099	0.89673	0.0109*
Male		1			

For all other dependent variables, no variables were retained in the models indicating there were no significant associations for these variables and participant characteristics.

Regarding readiness to change nutrition, gender was significantly associated. Females were more likely to be ready to change than males (OR = 1.14, CI = [1.13, 2.34], *p* = 0.0092).

For confidence to change physical activity, gender was also significantly associated.

Results indicated males being more likely than females to be highly confident to change (OR = 0.91, CI = [0.45, 0.99], *p* = 0.0109).

All other models did not retain any independent variables, indicating there were no significant associations between any of the participant characteristics and the variables: confidence for smoking, nutrition, alcohol and weight and readiness for smoking, alcohol, physical activity and weight.

## Discussion

This study provides data on five (SNAPW) lifestyle risk factors among CMHS clients across three regions of NSW Australia and is the first study, to the authors’ knowledge, that examines the relationship between participant characteristics and levels of readiness and confidence to make health behaviour change across these factors. A considerable proportion of participants were not meeting guidelines for lifestyle risk factors and many reported high readiness to change across risk factors. The proportion reporting high confidence to change was variable across risk factors, ranging from 32% for smoking to 68% for alcohol. Gender was associated with two dependent variables: with females more likely to be ready to change nutrition and males more likely to be confident in changing their physical activity.

Similar to other surveys of CMHS clients (Bartlem et al., 2015; Regan et al., 2022), this study showed substantial proportions of clients at risk for each SNAPW variable. Direct comparisons with risk levels in prior studies should be seen in light of differences in services, data collection tools and reporting of risks. Bartlem et al.’s (2015) study showed higher alcohol risk than the present study (43.2% vs 25.9%) and lower physical activity (46.8% vs 86.6%) risk. Regan et al. (2022) showed high levels of individual risks.

This study adds to the limited literature about the confidence and readiness of this population to make change, and characteristics associated with these outcomes. At least half of the participants felt ready to change most lifestyle factors, alcohol excepted. However, it was only for nutrition and alcohol use that at least half of the respondents felt highly confident about changing, with just below half for physical activity and weight, and around a third for smoking. Smoking showed the greatest discrepancy between readiness (67.9%) and confidence (32%). Smoking readiness rates in this study were higher than previously reported (Apodaca et al., 2007; Metse et al., 2016; Prochaska et al., 2014; Siru et al., 2009; Stockings et al., 2013). Low confidence levels pertaining to smoking were similar to previous research (Mann-Wrobel et al., 2011; Tzilos et al., 2014). Physical activity and weight showed a similar pattern, with more people ready to change than being confident to do so, though the discrepancies were smaller. Among people consuming alcohol, the proportion confident to make changes was greater than those who were ready to change (67.15%, 47.9%). Readiness levels were lower than in prior research (Prochaska et al., 2014). The pattern of results for alcohol could be contributed to by lower rates of risky drinking in this study (many of those asked about their confidence to change were drinking within recommended levels), and potential engagement with other services, such as alcohol and other drug services, or more global factors such as Australia’s cultural norms pertaining to drinking, and social acceptability (AIHW, 2024a; Bartram et al., 2017).

Significant findings regarding associations with gender should be interpreted in light of men typically being more physically active, and women more likely to be meeting nutrition guidelines (Hoare et al., 2018; Kanchi et al., 2018). Farholm and Sørensen’s (2016) systematic review of studies involving people with SMI found a relationship between gender and physical activity self-efficacy favouring men, with no association noted between gender and physical activity readiness. Although a significant association between smoking and gender was not found in the present study, previous research has noted a link between gender and smoking cessation, with females having increased success to quit (Prochaska et al., 2015).

This research adds support to policies and clinical practice guidelines (AIHW, 2022; Das et al., 2016; NSW Health, 2017; RANZCP, 2015) that recommend routine provision of preventive care within CMHS. Despite such guidelines, provision of care continues to be low (Bailey et al., 2019), with community mental health clinicians acknowledging several barriers (Fehily et al., 2023). One such barrier includes a perception that clients may have low desire to change health risks (Chwastiak et al., 2013; Robson et al., 2013; Wye et al., 2010), which contrasts the present findings. This demonstrates a need for further education and training to upskill mental health professionals in provision of preventive care, as well as for research which explores avenues to build client readiness and confidence. Such efforts may consider the broader contributors and social determinants of inequity, including low health literacy (Hassan et al., 2020), stigma and discrimination (Morgan et al., 2021a), lack of appropriate physical health services/interventions (Richardson et al., 2020), underpromotion and cost of services/interventions that do exist (Strunz et al., 2022), and unclear roles/responsibilities among health professionals (Laugharne et al., 2016). In addition to upskilling CMHS clinicians in delivery of preventive care, alternative approaches from previous research include the integration of a dedicated preventive care practitioner into mental health services which could see positive changes in readiness and confidence rates and overall preventive care (Fehily et al., 2020, 2022). Previous research has also discussed the potential role of peer workers in this space, given their ability to provide support informed by personal lived experience (Gill, 2012; NSW Health, 2021), with other methods including mobile applications (Schlosser et al., 2018), motivational interviewing (Romano and Peters, 2015) and consideration of biological underpinnings (Arnautovska et al., 2022).

This study has some limitations associated with the use of self-report data including issues with accuracy and social desirability bias, and individual conceptualisation of confidence and readiness variables. In addition, the readiness ruler was previously designed for alcohol, but was adapted by the team for other behaviours. Methodological constraints may impact generalisability of study findings, including the underrepresentation of people with higher SES, the exclusion of people who had only one appointment (criteria set for the larger trial) and restriction to three geographical regions in NSW. Associational analyses may not have explored all relevant independent variables due to limitations in data collection, e.g., the exclusion of some independent variables due to small cell counts; and lack of data on variables such as age of onset of mental health conditions and length of time in treatment. Regardless, this study provided data across multiple services and different geographical regions with a rigorous approach to analysis to determine associations, which may guide future initiatives to integrate preventive care into CMHS. More broadly, this study also adds to research and positional statements calling for improved efforts and/or reform to integrate physical health care into other services across the Australian health care system (Morgan et al., 2021b), e.g., community managed (non-government) organisations (Gibson et al., 2021).

## Conclusion

This research adds to limited literature pertaining to SNAPW risks in CMHS clients. It confirms high prevalence of these risks, with a considerable proportion of participants being ready and confident to change. Associations between participant characteristics and readiness and confidence show gender influencing nutrition readiness and physical activity confidence. Further research could focus on training to upskill clinicians in provision of preventive care that builds confidence and readiness levels for clients and takes opportunities to link clients to services to support change.

## Supplemental Material

sj-docx-1-anp-10.1177_00048674241257751 – Supplemental material for Risk prevalence, readiness and confidence to change lifestyle risk factors among clients of community mental health services Supplemental material, sj-docx-1-anp-10.1177_00048674241257751 for Risk prevalence, readiness and confidence to change lifestyle risk factors among clients of community mental health services by Tegan Stettaford, Caitlin Fehily, Elizabeth Campbell, Daniel Barker, Christopher Oldmeadow, Emma McKeon, Sophie Love, Sharon Lawn, David Castle and Jennifer Bowman in Australian & New Zealand Journal of Psychiatry

sj-docx-2-anp-10.1177_00048674241257751 – Supplemental material for Risk prevalence, readiness and confidence to change lifestyle risk factors among clients of community mental health services Supplemental material, sj-docx-2-anp-10.1177_00048674241257751 for Risk prevalence, readiness and confidence to change lifestyle risk factors among clients of community mental health services by Tegan Stettaford, Caitlin Fehily, Elizabeth Campbell, Daniel Barker, Christopher Oldmeadow, Emma McKeon, Sophie Love, Sharon Lawn, David Castle and Jennifer Bowman in Australian & New Zealand Journal of Psychiatry

sj-docx-3-anp-10.1177_00048674241257751 – Supplemental material for Risk prevalence, readiness and confidence to change lifestyle risk factors among clients of community mental health services Supplemental material, sj-docx-3-anp-10.1177_00048674241257751 for Risk prevalence, readiness and confidence to change lifestyle risk factors among clients of community mental health services by Tegan Stettaford, Caitlin Fehily, Elizabeth Campbell, Daniel Barker, Christopher Oldmeadow, Emma McKeon, Sophie Love, Sharon Lawn, David Castle and Jennifer Bowman in Australian & New Zealand Journal of Psychiatry
