# Supplementary figures and images for: Risk prevalence, readiness and confidence to change lifestyle risk factors among clients of community mental health services
Source: Aust N Z J Psychiatry. 2024 Jun 6;58(8):702–12. doi: 10.1177/00048674241257751 (PMC11308284; doi:10.1177/00048674241257751)

**Supplementary File 1**

Participant recruitment process


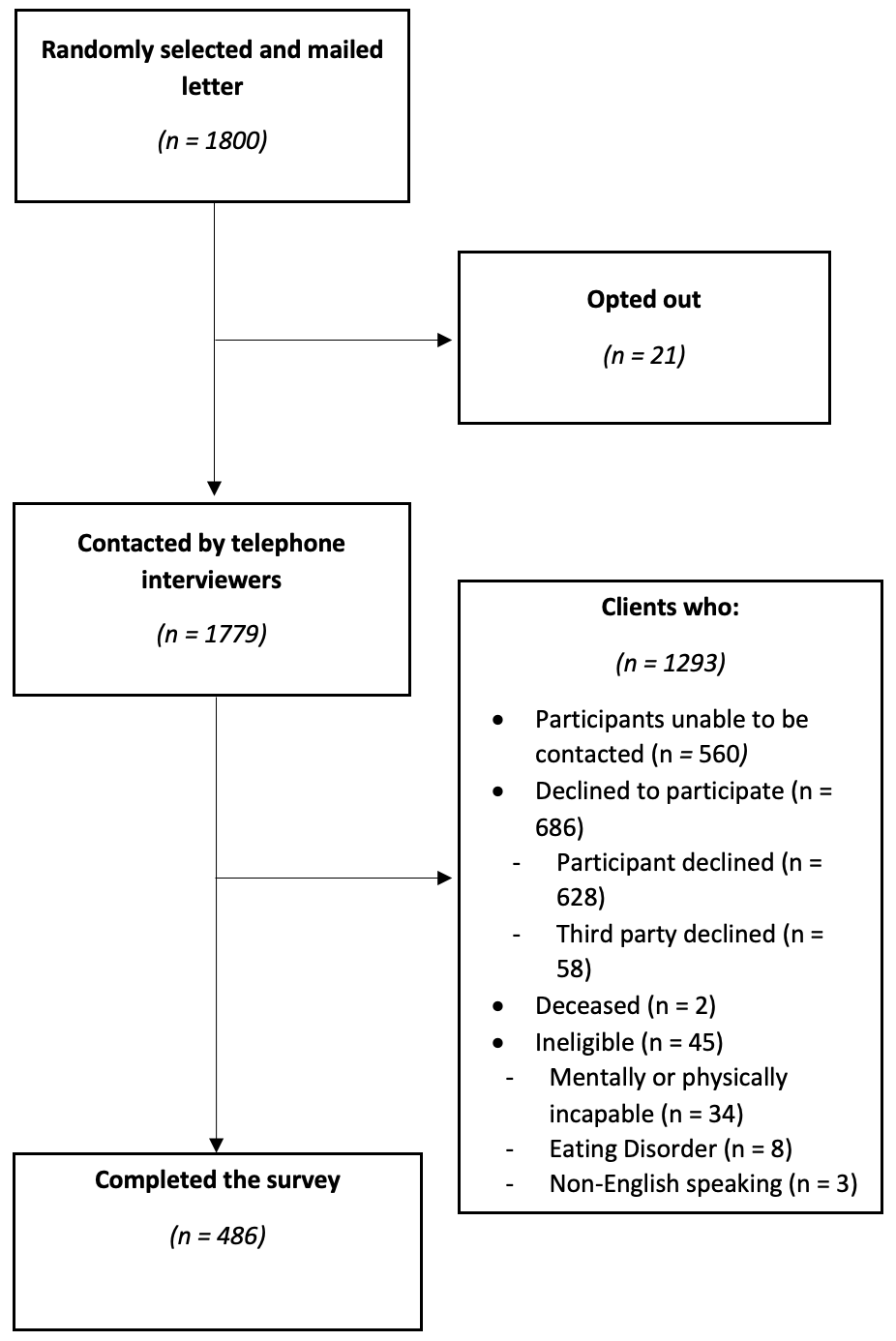

Supplement: sj-docx-1-anp-10.1177_00048674241257751 – Supplemental material for Risk prevalence, readiness and confidence to change lifestyle risk factors among clients of community mental health services [file sj-docx-1-anp-10.1177_00048674241257751.docx]
