# Supplementary material for: Risk prevalence, readiness and confidence to change lifestyle risk factors among clients of community mental health services
Source: Aust N Z J Psychiatry. 2024 Jun 6;58(8):702–12. doi: 10.1177/00048674241257751 (PMC11308284; doi:10.1177/00048674241257751)
Supplement: sj-docx-2-anp-10.1177_00048674241257751 – Supplemental material for Risk prevalence, readiness and confidence to change lifestyle risk factors among clients of community mental health services [file sj-docx-2-anp-10.1177_00048674241257751.docx]

**Supplementary File 2**

Associations between participant characteristics and confidence and readiness to change

|  |  | **AIC** | **OR** | **Lower CI** | **Upper CI** | ***p*** |
| --- | --- | --- | --- | --- | --- | --- |
| **Model 1: Smoking confidence (Backwards)** | | | | | | |
| *Remoteness* | | | | | | |
| Inner regional Australia | | 249.41 | 2.686182 | 1.074526 | 6.715109 | 0.0345* |
| Major cities of Australia | |  | 2.160278 | 0.811582 | 5.750288 | 0.1231 |
| Outer regional Australia | |  | 1 |  |  |  |
| **Model 2: Nutrition confidence (Backwards)** | | | | | | |
| *Age* | | | | | | |
| 18-34 | | 603.25 | 1.197368 | 0.682679 | 2.10009 | 0.5298 |
| 35-54 | |  | 1.663836 | 0.997952 | 2.774027 | 0.0509 |
| 55+ | |  | 1 |  |  |  |
| **Model 3: Alcohol Confidence (Backwards)** | | | | | | |
| *Remoteness* | | | | | | |
| Inner regional Australia | | 327.18 | 0.6155 | 0.282488 | 1.341086 | 0.2219 |
| Major cities of Australia | |  | 1.259824 | 0.527641 | 3.008014 | 0.603 |
| Outer regional Australia | |  | 1 |  |  |  |
| *Education* | | | | | | |
| No tertiary education | | 327.18 | 0.589615 | 0.346134 | 1.00437 | 0.0519 |
| Tertiary education | |  | 1 |  |  |  |
| **Model 4: Alcohol Confidence(Forwards)** | | | | | | |
| *Education* | | | | | | |
| No tertiary education | | 328.95 | 0.570103 | 0.33751 | 0.962992 | 0.0356* |
| Tertiary education | |  | 1 |  |  |  |
| **Model 5: Physical Activity Confidence (Backwards)** | | | | | | |
| *Remoteness* | | | | | | |
| Inner regional Australia | | 607.76 | 2.136833 | 1.211416 | 3.76919 | 0.0087* |
| Major cities of Australia | |  | 1.691027 | 0.933513 | 3.06323 | 0.0831 |
| Outer regional Australia | |  | 1 |  |  |  |
| *Gender* | | | | | | |
| Female | | 607.76 | 0.631345 | 0.422743 | 0.942876 | 0.0246* |
| Male | |  | 1 |  |  |  |
| *Marital Status* | | | | | | |
| Not partnered | | 607.76 | 0.646086 | 0.408244 | 1.022499 | 0.0622 |
| Partnered | |  | 1 |  |  |  |
| *Education* | | | | | | |
| No tertiary education | | 607.76 | 0.743842 | 0.495836 | 1.115899 | 0.1527 |
| Tertiary education | |  | 1 |  |  |  |
| *Mental Health Condition* | | | | | | |
| PTSD/Anxiety | | 607.76 | 0.464117 | 0.260782 | 0.825992 | 0.0091* |
| Depression/Bipolar | |  | 0.556237 | 0.341783 | 0.905245 | 0.0182* |
| Other | |  | 0.532577 | 0.277119 | 1.023533 | 0.0587 |
| Psychosis | |  | 1 |  |  |  |
| **Model 6: Physical Activity Confidence (Forward)** | | | | | | |
| *Remoteness* | | | | | | |
| Inner regional Australia | | 610.22 | 2.173872 | 1.245454 | 3.794376 | 0.0063* |
| Major cities of Australia | |  | 1.72584 | 0.964563 | 3.087958 | 0.066 |
| Outer regional Australia | |  | 1 |  |  |  |
| *Gender* | | | | | | |
| Female | | 610.22 | 0.59011 | 0.403855 | 0.862267 | 0.0064* |
| Male | |  | 1 |  |  |  |
| **Model 7: Weight Confidence (Backward)** | | | | | | |
| *Gender* | | | | | | |
| Female | | 590.40 | 0.666098 | 0.448014 | 0.990337 | 0.0447* |
| Male | |  | 1 |  |  |  |
| *Mental Health Condition* | | | | | | |
| PTSD/Anxiety | | 590.40 | 0.416877 | 0.236833 | 0.733792 | 0.0024* |
| Depression/Bipolar | |  | 0.640824 | 0.401206 | 1.023543 | 0.0625 |
| Other | |  | 0.509035 | 0.266572 | 0.972029 | 0.0408* |
| Psychosis | |  | 1 |  |  |  |
| **Model 8: Weight Confidence (Forward)** | | | | | | |
| *Gender* | | | | | | |
| Female | | 590.40 | 0.666098 | 0.448014 | 0.990337 | 0.0447* |
| Male | |  | 1 |  |  |  |
| *Mental Health Condition* | | | | | | |
| PTSD/Anxiety | | 590.40 | 0.416877 | 0.236833 | 0.733792 | 0.0024* |
| Depression/Bipolar | |  | 0.640824 | 0.401206 | 1.023543 | 0.0625 |
| Other | |  | 0.509035 | 0.266572 | 0.972029 | 0.0408* |
| Psychosis | |  | 1 |  |  |  |
| **Model 9: Nutrition Readiness (Backward)** | | | | | | |
| *Gender* | | | | | | |
| Female | | 617.11 | 1.540227 | 1.059132 | 2.239852 | 0.0238* |
| Male | |  | 1 |  |  |  |
| **Model 10: Nutrition Readiness (Forward)** | | | | | | |
| *Gender* | | | | | | |
| Female | | 617.11 | 1.540227 | 1.059132 | 2.239852 | 0.0238* |
| Male | |  | 1 |  |  |  |
| **Model 11: Alcohol Readiness (Backward)** | | | | | | |
| *Education* | | | | | | |
| No tertiary education | | 378.13 | 1.459545 | 0.904819 | 2.354363 | 0.1212 |
| Tertiary education | |  | 1 |  |  |  |
| **Model 12: Physical Activity Readiness (Backward)** | | | | | | |
| *Age* | | | | | | |
| 18-34 | | 582.60 | 2.001134 | 1.13636 | 3.524012 | 0.0163* |
| 35-54 | |  | 1.880232 | 1.136155 | 3.111609 | 0.014* |
| 55+ | |  | 1 |  |  |  |
| **Model 13: Physical Activity Readiness (Forward)** | | | | | | |
| *Age* | | | | | | |
| 18-34 | | 582.60 | 2.001134 | 1.13636 | 3.524012 | 0.0163* |
| 35-54 | |  | 1.880232 | 1.136155 | 3.111609 | 0.014* |
| 55+ | |  | 1 |  |  |  |
| **Model 14: Weight Readiness (Backward)** | | | | | | |
| *Age* | | | | | | |
| 18-34 | | 559.27 | 0.556161 | 0.30806 | 1.004078 | 0.0516 |
| 35-54 | |  | 1.051753 | 0.612026 | 1.807419 | 0.8551 |
| 55+ | |  | 1 |  |  |  |
| *Marital Status* | | | | | | |
| Not Partnered | | 559.27 | 0.492198 | 0.298153 | 0.812532 | 0.0056* |
| Partnered | |  | 1 |  |  |  |
| **Model 15: Weight Readiness (Forward)** | | | | | | |
| *Age* | | | | | | |
| 18-34 | | 559.27 | 0.556161 | 0.30806 | 1.004078 | 0.0516 |
| 35-54 | |  | 1.051753 | 0.612026 | 1.807419 | 0.8551 |
| 55+ | |  | 1 |  |  |  |
| *Marital Status* | | | | | | |
| Not Partnered | | 559.27 | 0.492198 | 0.298153 | 0.812532 | 0.0056* |
| Partnered | |  | 1 |  |  |  |
