# Supplementary material for: Risk prevalence, readiness and confidence to change lifestyle risk factors among clients of community mental health services
Source: Aust N Z J Psychiatry. 2024 Jun 6;58(8):702–12. doi: 10.1177/00048674241257751 (PMC11308284; doi:10.1177/00048674241257751)
Supplement: sj-docx-3-anp-10.1177_00048674241257751 – Supplemental material for Risk prevalence, readiness and confidence to change lifestyle risk factors among clients of community mental health services [file sj-docx-3-anp-10.1177_00048674241257751.docx]

**Supplementary File 3**

Summary of regression results: Client characteristics associated with readiness and confidence to change for the three regression methods

|  | ***Smoking*** | | | ***Nutrition*** | | | ***Alcohol*** | | | ***Physical Activity*** | | | ***Weight*** | | |
| --- | --- | --- | --- | --- | --- | --- | --- | --- | --- | --- | --- | --- | --- | --- | --- |
|  | ***BW*** | ***FW*** | ***LASSO*** | ***BW*** | ***FW*** | ***LASSO*** | ***BW*** | ***FW*** | ***LASSO*** | ***BW*** | ***FW*** | ***LASSO*** | ***BW*** | ***FW*** | ***LASSO*** |
| ***Gender*** |  |  |  | R* | R* | R* |  |  |  | C* | C* | C* | C* | C* |  |
| ***Age*** |  |  |  | C |  |  |  |  |  | R* | R* |  | R | R |  |
| ***Employment*** |  |  |  |  |  |  |  |  |  |  |  |  |  |  |  |
| ***Marital status*** |  |  |  |  |  |  |  |  |  | C |  |  | R* | R* |  |
| ***Education level*** |  |  |  |  |  |  | C R | C* |  | C |  |  |  |  |  |
| ***Remoteness*** | C* |  |  |  |  |  | C |  |  | C* | C* |  |  |  |  |
| ***Mental health condition*** |  |  |  |  |  |  |  |  |  | C* |  |  | C* | C* |  |

* Denotes significance (p<0.05); where not significant the variable was retained in the final model but p>0.05

C = Confidence

R = Readiness

LASSO = Least Absolute Shrinkage and Selection Operator

BW = Backward

FW = Forward
